# On the Relationships between Generative Encodings, Regularity, and Learning Abilities when Evolving Plastic Artificial Neural Networks

**DOI:** 10.1371/journal.pone.0079138

**Published:** 2013-11-13

**Authors:** Paul Tonelli, Jean-Baptiste Mouret

**Affiliations:** ISIR, Université Pierre et Marie Curie-Paris 6, CNRS UMR 7222, Paris, France; University of Vermont, United States of America

## Abstract

A major goal of bio-inspired artificial intelligence is to design artificial neural networks with abilities that resemble those of animal nervous systems. It is commonly believed that two keys for evolving nature-like artificial neural networks are (1) the developmental process that links genes to nervous systems, which enables the evolution of large, regular neural networks, and (2) synaptic plasticity, which allows neural networks to change during their lifetime. So far, these two topics have been mainly studied separately. The present paper shows that they are actually deeply connected. Using a simple operant conditioning task and a classic evolutionary algorithm, we compare three ways to encode plastic neural networks: a direct encoding, a developmental encoding inspired by computational neuroscience models, and a developmental encoding inspired by morphogen gradients (similar to HyperNEAT). Our results suggest that using a developmental encoding could improve the learning abilities of evolved, plastic neural networks. Complementary experiments reveal that this result is likely the consequence of the bias of developmental encodings towards regular structures: (1) in our experimental setup, encodings that tend to produce more regular networks yield networks with better general learning abilities; (2) whatever the encoding is, networks that are the more regular are statistically those that have the best learning abilities.

## Introduction

A major goal of bio-inspired artificial intelligence is to design artificial neural networks (ANNs) with abilities that resemble those of animal nervous systems [1]–[4]. A promising approach to design such “artificial nervous systems” is to use evolution-inspired algorithms, in particular because Darwinian evolution is regarded as the primary process responsible for shaping their natural counterparts. Despite the large amount of work in this direction, striking differences still separate most artificially-evolved networks from biological ones: biological nervous systems are *much larger* [5], *much more organized* [6], *much more plastic* [7] and, overall, much more complex [8].

It is commonly believed that the key for understanding the evolution of large and organized neural networks is the developmental process that links genes to nervous systems [1], [9]–[11]. The genotype of animals does not encode each synapse individually, it instead describes rules of development that are executed multiple times to give birth to networks with regular patterns of connection. Influenced by this concept, many researchers proposed *artificial developmental systems* with diverse inspirations including chemical gradients [11], [12], gene regulatory networks [13], [14], cell divisions [15], computational neuro-science models [16] and L-systems [9].

Nonetheless, most networks evolved with developmental systems cannot change during the “lifetime” of the controlled agent, whereas animal nervous systems are continuously changing to enable on-line behavioral adaptation and learning [7]. The basis of most of these changes seems to be provided by *synaptic plasticity*, that is, by the ability of synapses to strengthen or weaken over time [7], [17]. Several papers report experiments in which neural networks with synaptic plasticity are evolved [2], [15], [18]–[24]. Yet, only a handful of them use developmental systems [15], [20], [24].

The present paper shows that these two topics–developmental systems and synaptic plasticity–are actually deeply connected.

One of the main challenge when designing ANNs with learning abilities is to make them capable of learning in a large class of situations, that is, designing them so they can adapt their behavior to maximize a reward signal (or minimize an error) in as many situations as possible. For instance, it has been famously shown that single layer perceptrons are only capable of learning linearly separable patterns [25], whereas multi-layer perceptrons can learn any non-linear function (provided enough neurons are available) [26]. Single-layer perceptrons therefore possess lower learning abilities than multi-layer perceptrons: their architecture critically constrains what they can learn. When artificial evolution is used to design a plastic ANN, the topology of the networks is the result of the interactions between the fitness function, the encoding and the associated variation operators. As a consequence, the encoding and the fitness function have to be carefully crafted so that plastic neural networks are able to learn in as many situations as possible and, specifically, in situations that are not explicitly tested in the fitness function.

The most classic approach is to design a fitness function that tests each neural network in several test cases and rewards individuals that successfully adapt their behavior to each of them. To ensure that networks possess general learning abilities, it is then required to assess their abilities to learn in a new set of test cases, that is, test cases that have never been encountered by the evolutionary process [27]. The success of this “episodic fitness” approach relies on the assumption that if enough test cases are used, then it should become easier for the evolutionary process to design a generic structure than a specialized one.

Unfortunately, even in simplistic and constrained toy problems, the reported experiments show that many test cases need to be included in the fitness function to obtain general learning abilities. For instance [21], [23], [28], don't assess how evolved neural networks can cope with an unknown situation; counter-examples are [19] and [27]. (e.g. 10 to 20 test cases in [27]). For more complex problems, one can expect an exponential growth in the number of required test cases, because the number of possible test cases grows exponentially with the number of inputs/outputs. This approach is, therefore, unlikely to scale-up to life-like neural-networks.

**Figure 1:**
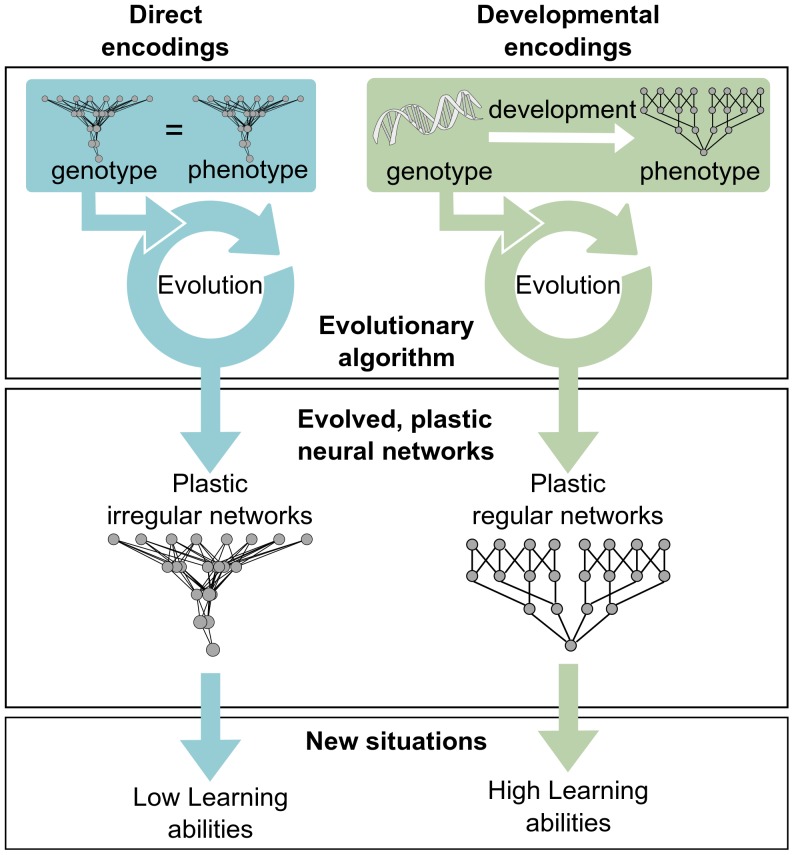
Main hypothesis. Using developmental encodings should facilitate the evolution of plastic ANNs with high learning abilities.

This is where developmental systems have a role to play. These systems evolve short descriptions of large structures by exploiting regularities observed in Nature, such as repetition of useful sub-parts, symmetries, and symmetries with variation [12], [29]. They more easily describe regular structures than irregular ones, because the former can be described by a few general rules whereas the latter require describing either each element, or a list of exceptions to general rules. As a consequence, developmental systems bias the search space towards regular structures [11]. *We here propose that this bias towards regularity is critical to evolve plastic neural networks that can learn in a large variety of situations* (Figure 1). Intuitively, this bias makes it more likely to obtain generic networks that apply the same learning rules to whole sets of inputs instead of networks that are finely-tuned to only solve the test cases used in the fitness function. A direct consequence is that *using developmental systems to evolve plastic neural networks should facilitate the evolution of plastic ANNs with general learning abilities*.

## Experimental Setup

**Figure 2:**
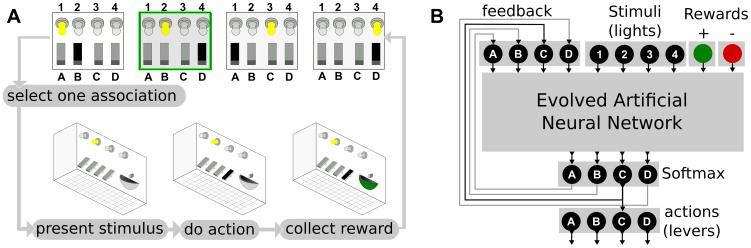
A. Concept of the “Skinner box”. A caged animal must learn to associate stimuli (here lights) to actions (here pushing a lever). The experimenter selects a stimulus/action association, presents it to the animal, record the action, and gives the reward to the animal. The experimenter then chooses another association in the association set and starts the cycle again. The association set is learned once the animal associates the right action to each stimulus. **B. Formalization of the Skinner box as a task for an artificial neural network.** Each stimulus is an input of the neural network. Positive and negative rewards are two additional inputs. The output is selected according to a softmax function (Methods) and the result of the softmax is looped back to the input layer.

This hypothesis is tested using a simulated “Skinner box” (Figure 2), a classic experimental setup for operant conditioning in which a caged animal must learn to associate stimuli (e.g. lights) to actions (e.g. pushing a lever). If the animal executes the correct action, it is rewarded (e.g. by some food); if it chooses the wrong one, it is punished (e.g. by an electric shock). There is no delay in the reward, so there is no credit assignment problem [30]. We consider only one-to-one associations so that, for each simple stimulus (each light), there is a different action to perform. Four stimuli and four actions are used; there are therefore 256 possible sets of stimulus/action (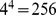; see Appendix S1 for the list of possible association sets). We formalize the stimulus/action associations using the concept of association sets:

**Definition 1 (Association):** *An association is a pair* 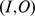 *of input/output that leads to the maximum positive reward. In our system (* 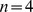 *:* 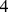 *inputs,* 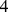 *outputs),* 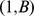 *is an association that means that the agent must push the* 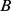 *lever when light* 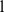 *is on.*

**Definition 2 (Association set):** *An association set* 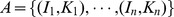 *is a list of associations that covers all the* 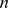 *possible inputs. For instance, the list of associations* 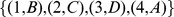 *is an association set in our system (* 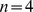 *:* 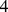 *inputs,* 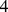 *outputs). Several inputs can be associated to the same output. For instance, the association set* 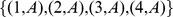 *is also valid in our system.*

**Definition 3 (Global training set):** *The global training set, called* 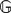 *, is the the set of all the possible association sets of an experimental setup. In our system, there are* 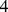 *possible outputs and* 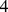 *possible inputs (* 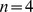 *), therefore the size of* 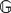 *is* 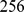 *(* 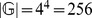 *; the complete list of association sets is available in File S1). The ideal plastic network should be able to learn every association sets of* 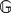 *.*

The fitness function (Methods) assesses the ability to learn a subset of the global training set, called the *evolutionary training set*:

**Definition 4 (Evolutionary training set):** *The evolutionary training set, called* 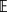 *, is the set of the association sets used in the fitness function.*


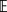
 is included in 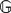; it does not change during an experiment. Depending of the experiment, the size of 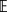 varies between 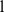 and 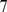. The elements of 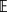 have been chosen at random.

The fitness function is normalized by the size of 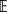, so that it actually corresponds to the the number of successfully learned sets divided by 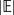. After each evolution experiment, we assess the ability of the network with the best fitness score to learn every possible association set, that is, we evaluate the fitness function on the global training set. We call the success rate of this test the *General Learning Abilities score (GLA score)*. This score reflects how well networks that are selected for their capacity to learn a few association sets are able to learn association sets that have not been encountered during evolution.

The evolved ANNs (Figure 2, B) have one input for each possible stimulus (i.e., 4 stimuli inputs), one input for positive rewards and one input for negative rewards. They have 4 outputs, each of them representing the probability of choosing each action. The final action is selected thanks to a “softmax” function that randomly selects an action according to a distribution that gives a higher probability to actions that corresponds to high output values distribution [30] (Methods). In effect, the neural network can activate any combination of the four available outputs and the softmax function makes sure that only one action is chosen at a time (Figure 2, B). Only one light (input) is activated at a time.

Plasticity is implemented in the neural networks using *neuro-modulated Hebbian plasticity* [2], [7], [21], [23] (Methods). In this model, neurons are of two kinds, “standard” and “modulatory”; the strength of connection between each pair of neurons is modified using a classic Hebbian rule that is activated only if the sum of inputs from modulatory neurons is above an evolved threshold.

For each association of 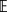, the fitness function first presents the stimuli to the neural network for a few time-steps (Methods). Once the final output is computed by the softmax, it is copied to the input layer (feedback inputs). The reward input (positive or negative) is set at the same time. Such feedback loops are often present in computational models of cortex-basal ganglia-thalamus-cortex loops for action selection [31]–[33] and are implicit in actor-critic models of reinforcement learning [30]. The neural network is then simulated for a few more time-steps (Methods). It is expected that the evolutionary process will connect one or several modulatory neurons to the reward input and that the ANNs will exploit the copied outputs to strengthen/weaken the connections that correspond to the action that has actually been performed. Nonetheless, it must be emphasized that weight changes can occur at any time, including during the first step of the evaluation of the ANN. Only the topology and the synaptic weights of the ANNs, which are designed by evolution, determine when and how synaptic weights change.

The ANNs that solve this task may seem trivial at first sight. However, the evolutionary process needs to add at least one modulatory neuron (inputs cannot be modulatory in our system) and we never found any solution with less than two hidden neurons (one of them being modulatory). Essentially, the challenge raised by this task is to discover learning rules that allow the ANN to exploit a reward to strengthen and weaken the right connections. Typical solutions require three main “discoveries”: (1) identifying and correctly connecting the reward inputs, (2) gating the reward with the softmax choice to modify only the connections corresponding to the chosen action, and (3) applying the resulting reinforcement to a link between the inputs and the output.

**Figure 3:**
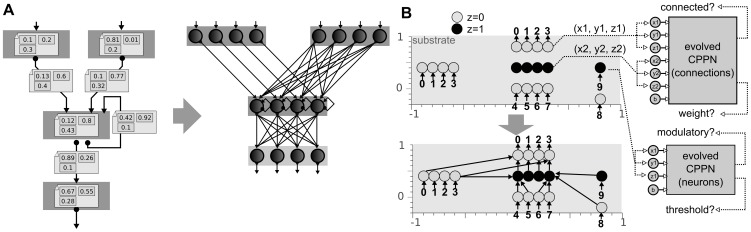
A. Principle of the map-based, developmental encoding. The neural network is encoded as a labeled graph (left), which is developped to a graph of maps according to the labels (right). (Methods). **B. Principle of the HNN encoding (minimal HyperNEAT).** Neurons are placed in a 3D substrate (top). To know whether two neurons are connected and the synaptic weight of each connection, a Compositional Pattern Producing Network (CPPN) is queried using the 3D coordinates of the two neurons (Methods). This CPPN is evolved using a direct encoding. To know the parameters of each node (neuron type and threshold value), a second CPPN is queried with the 3D coordinates of the neuron (Methods).

The topology and the parameters of evolved ANNs are encoded with three encodings [2], with three different levels of expected regularity (Figure 3). The first encoding, called the map-based encoding [16] (Methods), is inspired by computational neuroscience models in which ANNs are described as graph of single neurons and *neural maps* (spatially-organized identical neurons) that are connected with a few possible connection schemes (usually only one-to-one and one-to-all) [33]–[35]. This encoding produces very regular neural networks because it has to treat each neuron in a map in the exact same way as the other neurons of the same map. The second encoding is a simplified version HyperNEAT [12], called HNN, for Hyper Neural Network (Methods). HyperNEAT-like encodings are developmental encodings in which morphogen gradients are described as feed-forward networks of mathematical functions that operate in a Cartesian space. This indirect approach allows them to encode large networks with Nature-like connection patterns (symmetry, symmetry with variations, repetition, etc.). The last encoding is a classic direct encoding in which evolution directly acts on the structure and the parameters of the ANN (Methods). This encoding has no bias to produce regular networks.

To understand the relationship between encodings, regularity and learning abilities, we have to assess the regularity of evolved ANNs. According to Lipson [36], regularity is the compressibility of the description of the structure. Regrettably, this value is not computable [37] and, to our knowledge, there exists no well-recognized approximation for weighted, directed graphs. The few algorithms designed to compress the graph structure are greedy approximations that only work well for sparse, undirected labeled graphs [38], [39]. We follow another method to estimate the regularity of networks: counting the number of symmetry axes [40]–[42]. A graph has an axis of symmetry when two groups of nodes can be swapped without modifying the graph, that is, when there is a repetitive, structural pattern. More axes of symmetry means a better compression because the two groups need to be described only once [40]–[42]. In graph theory, this kind of symmetry is called an automorphism and fast algorithms exist to count them [43]–[45] (Methods).

Networks are evolved using the classic multi-objective evolutionary algorithm NSGA-II [46], [47]. Two objectives are optimized: the fitness of networks (Methods) and a behavioral novelty objective [4], [22], [23], [48], [49], to mitigate premature convergence (Methods). These two objectives are optimized during a maximum of 4000 generations of 400 individuals. Experiment are stopped as soon as the best individual of the population reaches a perfect fitness value on the evolutionary training set. At the end of each experiment, the novelty objective is discarded and we consider that the best individual is the one with the best fitness value.

We perform 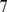 series of independent experiments by varying the size of the evolutionary learning set from 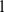 to 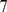 (i.e., 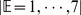). For each series, the three investigated encodings are tested (direct encoding, map-based encoding and HNN encoding). Each experiment is replicated 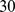 times to obtain statistics. We therefore launch a total of 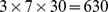 experiments, each one lasting between 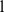 and 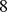 hours on our computers (Intel Xeon E5520@2.27 GHz) depending on the time required to converge and the size of the evolutionary training set. Because of this large computational time, we were not able to extend our experiments to harder problems, for instance with more inputs/outputs.

## Results

For each encoding, we compute the GLA score of networks with a perfect fitness on the evolutionary training set and we plot it as a function of the size of the evolutionary training set.

**Figure 4:**
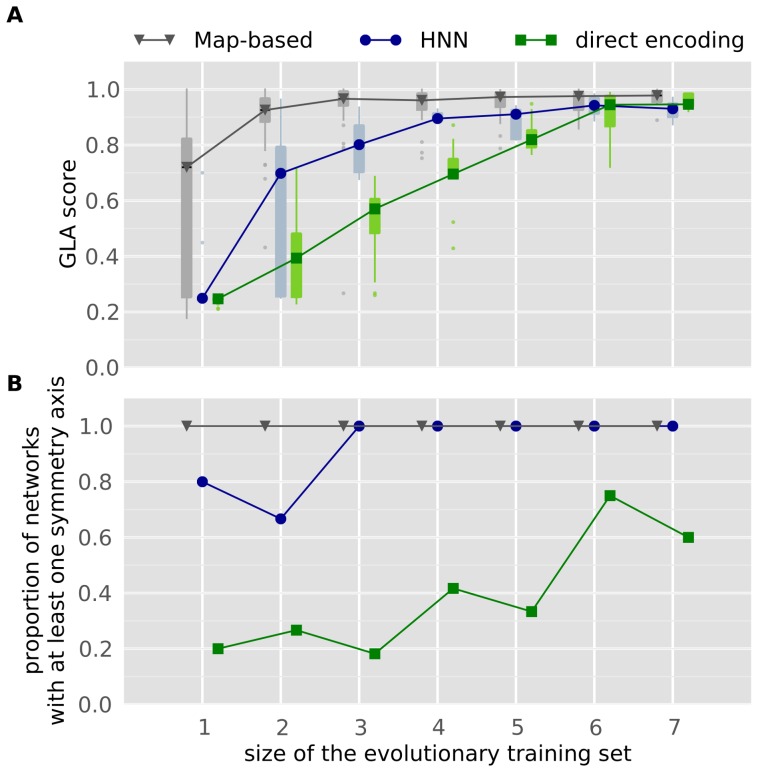
Relationship between encodings, general learning abilities and the size of the evolutionary training set (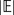). **A**. Generative encodings yield plastic ANNs with better general learning abilities than those evolved with a direct encoding. Morever, increasing the size of 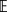 increases the general learning abilities. Each box extends from the lower to upper quartile values of the data, with a symbol at the median. Whiskers extend to the most extreme data point within 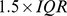, where IQR is the interquartile range. Flier points (outliers) are those past the end of the whiskers. X-values are shifted for the map-based encoding and the direct encoding in order to make the figure readable. **B**. Generative encodings yield more regular networks than a direct encoding, and increasing the size of 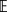 increases the regularity of evolved networks.

The results show a clear difference in the GLA scores obtained with each encoding (Figure 4, A). With a direct encoding, the GLA score grows linearly with the size of the evolutionary training set, which is consistent with previous results [27], and the GLA scores obtained with small values of 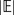 are statistically different from those obtained with larger values (e.g., 1 versus 7: 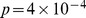; 4 versus 7, 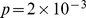, 3 versus 6, 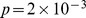; unless otherwise specified, the statistical test in this paper is the Mann-Whitney U-test). With the direct encoding, using a fitness that tests at least 6 associations sets (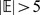) is required to obtain networks with a GLA-score similar to the one reached with the map-based encoding with only 2 association tests (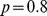). The HNN encoding appears as a trade-off between the direct encoding and the map-based encoding: for each value of 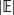, the GLA score obtained with HNN is consistently higher than the one obtained with the direct encoding, yet it is lower than the one reached with the map-based encoding (for 2, 3 or 4 association sets, HNN versus direct encoding, 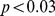; for 1, 2, 3 or 5 association sets, HNN versus map-based encoding, 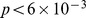; with 4 association sets and the HNN encoding, there are not enough networks with a perfect fitness score to perform a statistical analysis).

As expected, each encoding leads to different levels of regularity, and increasing the number of association sets used in the fitness function increases the regularity of evolved neural networks (Figure 4, B). All the networks evolved with the map-based encoding are regular: they all have at least one symmetry axis. The HNN encoding also leads to many networks with at least one symmetry axis (from 80% to 100%), whereas the direct encoding leads to substantially fewer regular networks (from 20% to 70%, depending on 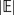). These numbers vary with the size of 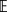. With the HNN encoding, three association sets are needed to obtain 100% of regular networks; with the direct encoding, the number of regular networks grows from 20%, when one association set is used during evolution (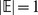), to 60–70% when more than 6 association sets are used (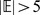).

**Figure 5:**
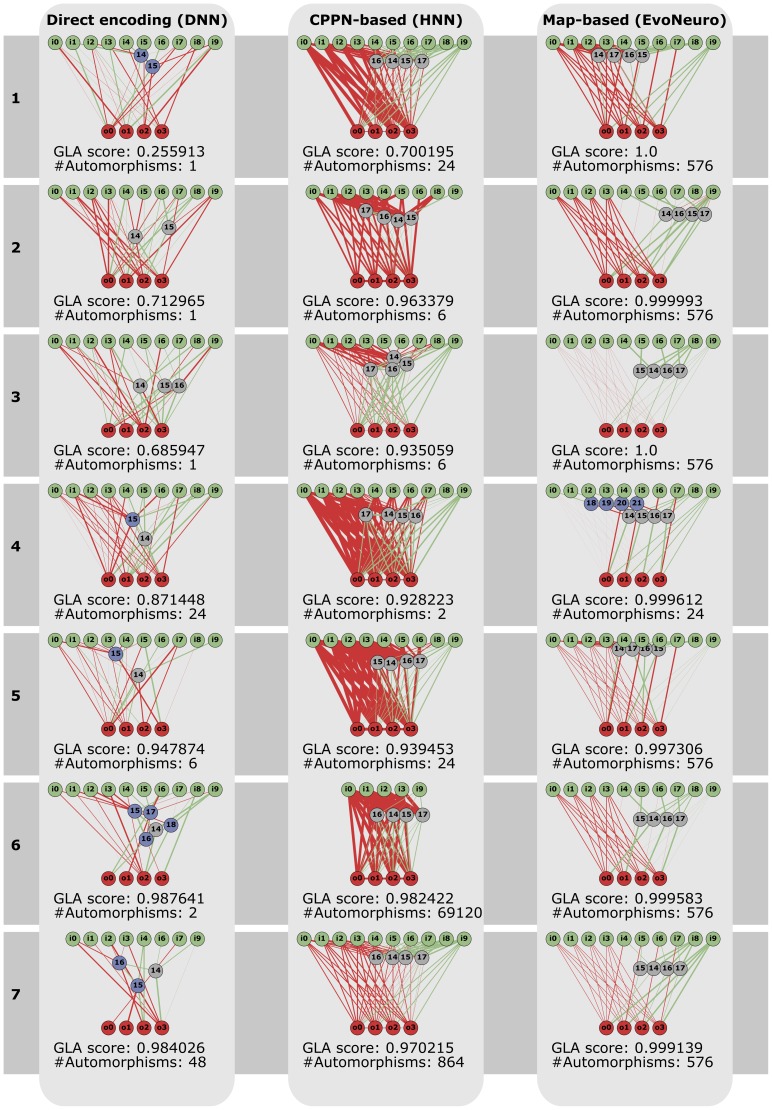
Network with the best learning abilities, for each encoding and each size of the evolutionary learning set. Each network is the best (in term of learning abilities) of the 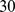 independent runs. Inhibitory connections are represented as green line and excitatory ones as red lines. The width of the lines is proportional to the corresponding synaptic weight (for modulated connections, the line width is determined after one of the learning phases, for one of the possible association sets). Input neurons are in green, output neurons in red, modulatory neuron in gray and standard neurons in blue. “#automorphisms” means “number of automorphisms” (Methods). Nodes that are not connected (directly or indirectly) to at least one input and one output are not drawn.

To further understand this result, we plot the network with the best learning abilities for each encoding and each size of the evolutionary learning set (Figure 5). We observe the same overall link between learning abilities and regularity as on Figure 4, but some networks have good learning abilities with only a few automorphisms, like the network evolved with a direct encoding and 6 association sets (GLA score of 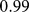, 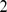 automorphisms). This result is possible because nothing encourages a directly encoded network to duplicate the same sub-structure several times: it may be sometimes easier to either re-invent 4 times the same function but with slight changes, or to design an integrated solution that relies on only one complex structure. This particular network seems to use a centralized structure with only one modulatory neuron that modulates all the plastic connections of the network. Conversely, some regular networks have a low GLA score, such as the network evolved with HNN and one association set (GLA score of 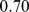, 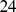 automorphisms). There is no paradox in this result: the regularities can be at the wrong place to lead to high-learning abilities.

**Figure 6:**
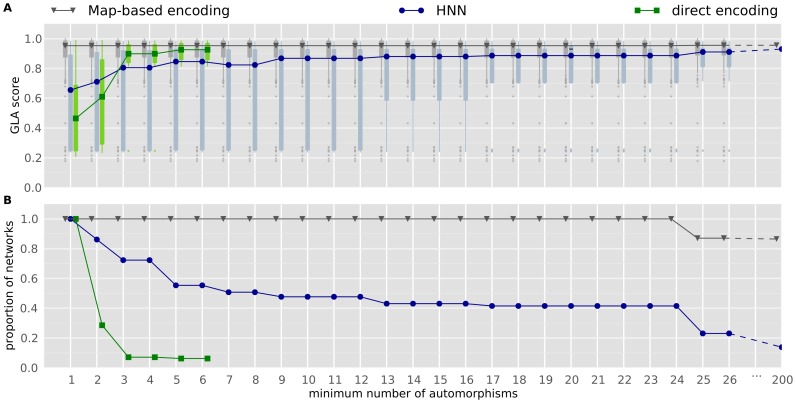
Relationship between regularity and general learning abilities. Data are from the same experiments as Figure 4. The “minimum number of automorphims” means that if, for example, a network has 4 automorphisms, it is included in columns 1,2,3 and 4. X-values are shifted for the map-based encoding and the direct encoding in order to make the figure readable. **A**. The more automorphisms a network has, the more likely it is to have good general learning abilities (GLA score). Each box extends from the lower to upper quartile values of the data, with a symbol at the median. Whiskers extend to the most extreme data point within 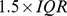, where IQR is the interquartile range. Flier points (outliers) are those past the end of the whiskers. **B**. 7% of networks evolved with a direct encoding have more than 3 autormorphisms. 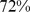 of those evolved with HNN have more than 3 automorphisms. 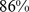 of networks evolved with the map-based encoding have more than 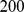 autmorphisms; 100% of them have at least 10 automorphisms.

Whatever the encoding and the size of 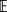 are, networks with the best learning abilities are those that are the most regular (Figure 6, A; this figure use the same data as Figure 4). Hence, among networks evolved with the direct encoding, those that have at least 2 automorphisms (one axis of symmetry) have a better GLA score than those that have no automorphism (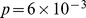). Those with more than 3 automorphisms also have statistically better learning abilities than those with two automorphisms (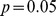) and than those without any symmetry axis (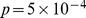). The same tendency is present with the HNN encoding: networks with at least two automorphisms (i.e., networks with at least one symmetry axis) have a higher GLA score than those that have no symmetry axis (one automorphism, 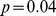); networks with more than 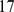 automorphisms have a higher GLA score than those with at least two automorphisms (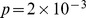).

With the HNN encoding, 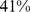 of networks have exactly 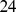 automorphisms but only 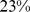 of them have 25 or more automorphisms (Figure 6, B, blue line). With the map-based encoding, a drop from 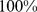 to 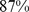 occurs at the same number of automorphisms (Figure 6, B, grey line). A network with 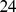 automorphisms is a network in which a sub-network is repeated 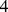 times (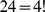, Methods). This number is particular in our experiments because both HNN and the map-based encoding group neurons by 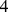, therefore the number of automorphisms is expected to be a multiple of 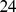: a different number means that at least one neuron of a group has a connectivity pattern that is different from the rest of the group. With HNN, this kind irregularity is possible but unlikely. With the map-based encoding, it is not possible, that is why all map-based networks have a number of automorphisms exactly equals to a multiple of 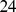 (for instance, on figure 5, all map-based networks have 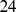 or 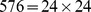 automorphisms).

## Conclusion and Discussion

The experiments reported in this paper add weight to the hypothesis that using a developmental encoding improves the learning abilities of evolved, plastic neural networks. Complementary experiments reveal that this result is the consequence of the bias of developmental encodings towards regular structures [11]: (1) encodings that tend to produce more regular networks yielded networks with better general learning abilities; (2) in our experimental setup, whatever the encoding is, networks that are the more regular are statistically those that have the best learning abilities. This second point implies that an indirect encoding that is not biased towards regular network should not lead to ANNs with high learning abilities; it also implies that a direct encoding combined with a helper objective that encourages regularity should lead to ANNs with good learning abilities (see [4] and [50] for examples of helper objectives with a direct encoding). Nonetheless, our experiments show that current generative encodings and neuro-modulated Hebbian plasticity make a promising combination to evolve large, plastic neural networks. Future work in this direction should investigate whether this combination holds its promises in other tasks such as learning in a maze [21], [23] or visual processing [12].

According to our results, neural networks evolved with an encoding biased towards regularity could be more flexible than those evolved with an unbiased encoding: they are better at learning association sets that have never been encountered during their evolution. To achieve this flexibility, they have to possess connections that were not directly selected during evolution. In other words, their flexibility stems from “spandrels” [51]: they are the byproducts of the bias that make evolution more likely to duplicate a sub-structure than to design a specialized circuit.

These results are in opposition to the general tendency of neural networks to minimize connection costs [50], [52]–[54] because they show that flexible behaviors require maintaining many “useless” connections. They indicate that a selective pressure for flexibility is likely to favor developmental procedures that would result in connections that do not procure any short-term advantage. In a constant environment, these connections should disappear; but in a constantly changing environment – which puts more pressure on flexibility –, these connections appear critical. This view is consistent with the theory of “variability selection”, which posits that flexibility is one of the primary selective pressure that shaped the brains of hominids [55], [56].

The conflict between flexibility and connection costs also echoes the debate about the modularity/non-modularity of the mammalian brain [6], [57], [58], since the minimization of connection costs has been linked with the evolution of modularity [50], [59]. Our results thus suggest that the parts of the brain that heavily rely on synaptic plasticity to achieve flexible behaviors should be less modular than simpler, less plastic parts. To test this proposition, it is possible to launch computational experiments in which plastic neural networks are evolved with a selective pressure to minimize connection costs and different flexibility requirements.

Pushed to the extreme, the results of our experiments suggest that the best flexibility would be achieved with fully connected networks, since this would be the best possible regularity. In real brains, such a connectivity would be challenging for pure physical reasons [5], [52]: if each neuron of a mouse was connected to each other, its brain (about 10 millions neurons) would at least occupy 350 cubic meters [51] (about the cranial volume of an Orangutan). Artificial brains do not have such limitations and can be designed as fully connected [60], but most neural networks used in machine learning are made of layers of neurons, with each layer fully connected to the next one [26], [61]. Layers are a very specific structure that prevents some flexibility (non-Markovian tasks cannot be learned), but they make learning easier, because feed-forward networks have no intrinsic dynamics (contrary to recurrent neural networks). These networks are still very regular and flexible. In image processing, convolutional neural networks are classic feed-forward neural networks in which many, well-chosen connections are removed and many synaptic weights are constrained to be equal [62]. These networks are much easier to train than classic layered neural networks, but they cannot learn when the input data do not look like images.

These examples highlight a potential trade-off between flexibility and trainability, or, put differently, between learning abilities and learning efficiency: in many situations, it seems beneficial to trade some flexibility to make the system easier to train. Our experiments considered a simple situation in which trainability was not a major concern because the input/output patterns are simple and low-dimensional. In more challenging tasks, the evolutionary process would probably have to find the best trade-off between trainability and flexibility, and therefore between regularity and specialization. Nonetheless, although convolutional networks are less regular than multi-layer perceptrons, they are still very regular and could be generated with a generative encoding. Generative encodings that aim at intermediate regularity might thus be one of the key to explore this trainability/flexibility trade-off.

Overall, the present paper shows that evolution, development and synaptic plasticity are three interleaved processes that are hard to study separately. While an extensive understanding of their interactions is probably out of reach with the current state of knowledge, studies that combine simple models of each of these processes shed light on how one of them – here development – can simplify another – here learning. Such studies appear helpful for both building a global vision of the evolution of intelligent lifeforms as well as harnessing evolution to create intelligent agents.

## Methods

### Plastic neuron model

Following [21]–[23], we distinguish two types of neurons: “standard neurons” and “modulatory neurons”. Inputs of each neuron are divided into modulatory inputs 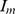 and standard 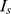 inputs. The output 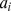 of a neuron 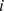 is then defined as follows:
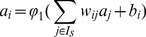
(1)where 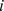 is the identifier of a neuron, 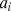 its output, 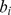 its bias, 
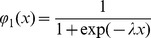
 a sigmoid on 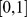, 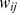 the synaptic weight between neurons 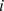 and 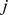. Each non-modulatory synaptic weight 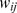 is modified with regards to the sum of modulatory inputs and a constant coefficient 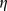 (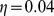 in our experiments):
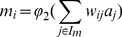
(2)

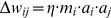
(3)

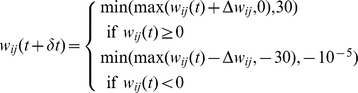
where 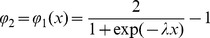 is a sigmoid on 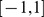 (to allow positive and negative modifications of synaptic weights).

### Fitness function and behavioral descriptors

The fitness function computes the number of associations that the network successfully learn, given an evolutionary learning set 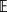.

For each association set of 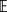 (for instance, 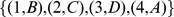), this function first randomly initializes the modulated weights, that is, the network does not have to un-learn what was previously learned. The network is then allocated 90 learning episodes, each one executing the following steps:

- successively select one of the four associations of the association set (for instance, 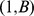);
- set the stimuli inputs of the neural network according to the chosen association and set the other inputs (reward and feedback) to zero (for instance, for the association 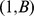, the input will be 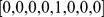);
- compute the output of the network by simulating it during 5 time-steps (5 time-steps is enough to allow a signal to travel from the inputs neurons to the output neurons);
- select an action using the four outputs of the neural network and a softmax function (see section “Output selection”);
- set the reward inputs (i.e., positive reward if the output is correct, negative otherwise) and the feedback inputs (for instance, if the output is “C”, a wrong answer, then the new input will be 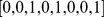);
- simulate the network again for 5 time-steps (this is the step where the network is expected to reinforce connections; however, nothing prevents adaptation to occur during the previous activation);
- if the last learning episode for this association set is reached:
  the positive reward that corresponds to each input of the set is added to the fitness (for instance, if the network's output was 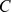 for association 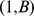, 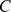 for 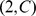, 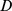 for 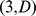 and 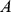 for 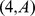, then 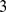 is added to the current value of the fitness);
  the output of the network (before the softmax) for each input of the set is appended to the behavior descriptor (for instance, for the previously described outputs, we would append the vector 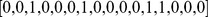);

In summary, the final fitness value corresponds to the average number of associations of 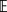 that have been successfully learned. The final behavior descriptor is a vector that contains the final output of the network for each association of 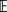.

**Figure 7:**
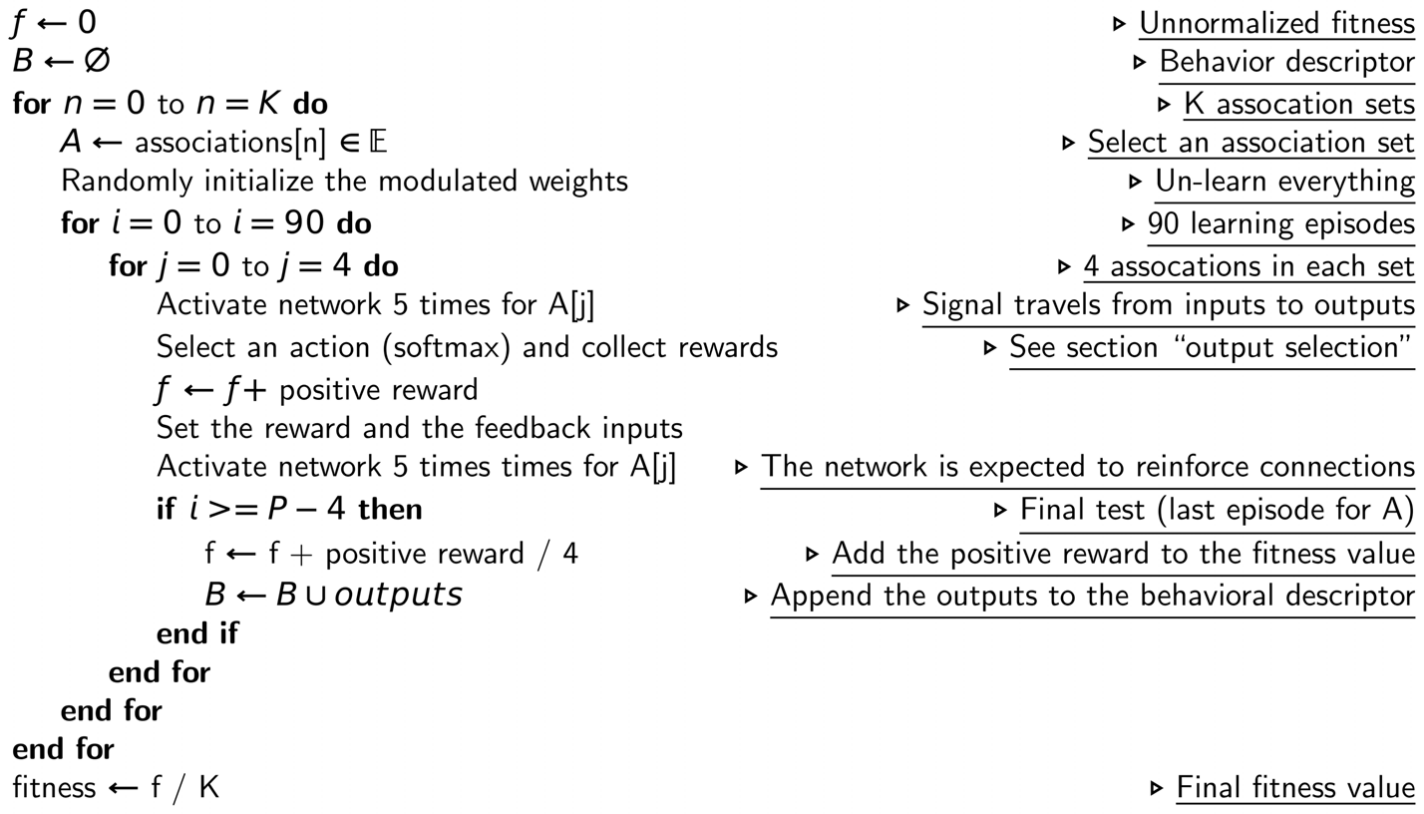
Algorithmic view of the fitness function.

This fitness function is described in a more algorithmic on figure 7.

### Output selection (softmax)

At the ouptut of the neural networks, the action is selected thanks to a softmax function [30]:
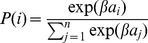
(4)where 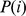 is the probability of selecting output 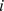, 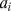 is the activity of output 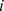 and 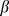 is a constant. In effect, this distribution gives a higher probability to actions that corresponds to high output values. If values are close, then they will have similar chances to be selected; if values are very contrasted, then the softmax function is equivalent to a “max” function. Using this distribution instead of a simpler “max” function allows network to explore – which is required to learn – and encourages the contrast between output. This technique is commonly employed in reinforcement learning [30].

### Map-based encoding

Many computational neuroscience models (e.g. [33]–[35]) are described as graph of neural maps (spatially-organized identical neurons) in which each connection is labeled by a set of parameters that represent the connection scheme [16](Figure 3). This description of neural networks can be seen as a developmental encoding according to which networks of maps are developed to form a neural networks.

In our model, each edge is associated with three parameters: (1) connection type (“1 to 1” or “1 to all” with uniform synaptic weights); (2) synaptic weight (all connections between maps have the same strength) (3) inhibitory or excitatory (a Boolean value). Similarly, three parameters describe each map: (1) isolated neuron or map of neurons (a Boolean value); (2) inhibitory or excitatory (a Boolean value – the whole map will be inhibitory or excitatory); (3) parameters of the neuron (float number, threshold value). Each label is encoded with a real number in 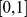, mutated with polynomial mutation in the same way as intrinsic parameters of neurons and synaptic weights in a direct encoding. The section “parameters” describes how these numbers are translated into Boolean values and parameters.

In the present study, all maps have the same size. Each graph of neural maps is developped into a full neural network by analyzing each node and each edge of the graph to create the corresponding neurons, maps and connections.

Labeled graphs are evolved using the direct encoding (Methods).

### HNN encoding (minimal HyperNEAT)

HyperNEAT is a developmental encoding in which morphogen gradients are described as feed-forward networks of mathematical functions that operate in a Cartesian space [12], called Compositional Pattern Producing Networks (CPPNs). When evolving an ANN, HyperNEAT evolves CPPNs that are then *queried* to know each synaptic weight. This indirect approach allows HyperNEAT to evolve large networks with Nature-like connection patterns (symmetry, symmetry with variations, repetition, etc.).

In the present study, we use a simplified version of HyperNEAT in which the CPPNs are evolved using a simple direct encoding instead of the NEAT method. We chose this simplified version to make easier the reproduction of our results, to enable the use of multi-objective evolutionary algorithms and to focus our study on the developmental process. We call this encoding HNN (Hyper Neural Network).

We place 9 input neurons, 5 hidden neurons and 4 output neurons in a 3D substrate (Figure 3, B). We describe each individual with two CPPNs: one connection-centred CPPN that returns whether a connection exists (LEO link in HyperNEAT [63]) and the synaptic weigth, and one node-centred CPPN that returns the intrinsic parameters of each neuron, that is, the threshold value used in the sigmoid and whether the neuron is “modulatory” or “standard”. Three functions are available to the CPPNs: 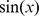, 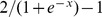 (sigmoid), 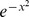 (Gaussian) and 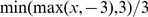 (linear).

Many other substrates can be used and some of them undoubtedly lead to faster convergence; it is also possible to use a single CPPN for both the connection and the node parameters. Nevertheless, the present work is centered on the *consequences* of using *any* developmental encoding when evolving plastic neural networks. The relative performance of each encoding is irrelevant.

### Direct encoding (control experiment, labeled graph, CPPN)

We use a straighforward direct encoding, loosely inspired by NEAT [64], to encode both the labeled graph of the map-based encoding and CPPN for the HNN experiments. We also use it as a control experiment. In this case, the evolved graph is employed in a more classic fashion to directly define a neural network.

In this encoding, a neural network (or a CPPN) is described as a directed graph and five mutation operators are implemented:

- add a connection between two randomly chosen neurons (probability: 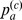);
- remove a randomly chosen connection (probability: 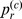);
- move the target or the source of a randomly chosen connection (probability: 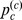);
- add a neuron by splitting an existing connection in two (the connection weight is kept on the two connections) (probability: 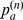);
- delete a randomly chosen neuron and all related connections (probability: 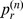);
- change random weights using the polynomial mutation [46] (probability: 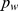 for each connection);
- change the intrinsic parameter of a neuron (e.g. the activation function when evolving CPPNs) (probability: 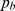 for each neuron);.

Cross-over is not employed. To initiate the evolutionary process, neural networks of the first generation are feed-forward networks without hidden layer, each one with randomly generated weights. This encoding has been previously employed in many papers, with similar parameters [4], [49], [65]–[67].

### Counting automorphisms

**Definition 5 (Automorphism):** *An automorphism of a graph* 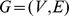 *is a permutation* 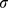 *of the vertex set* 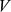 *, such that the pair of vertices* 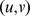 *forms an edge if and only if the pair* 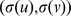 *also forms an edge. Put differently, an automorphism is a graph isomorphism from* 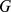 *to itself.*

**Figure 8:**
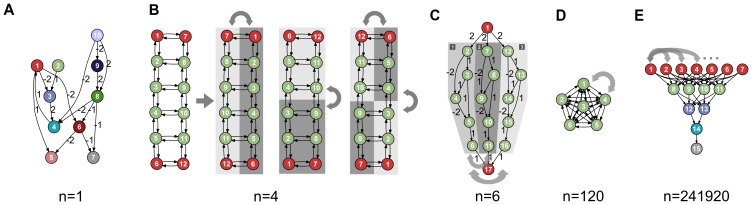
Examples of networks and corresponding number of automorphisms. (Colors are only here to help seing the symmetry axes, they have no particular meaning). **A**. A random network typically has 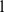 automorphism (itself). **B**. The central pattern generator of the lamprey [68] has 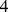 automorphisms (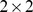) because it has two axial symmetries: top-down and left-right. The structure of the graph implies that the vertex orderings 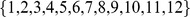, 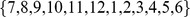, 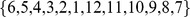 and 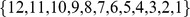 all lead to the same connectivity matrix. **C**. This network has 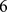 automorphisms (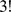) because modules marked 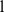, 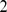 and 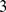 can be swapped without changing the connectivity of the network. **D**. This fully connected network with uniform synaptic weights has 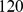 automorphisms (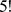) because each of its nodes can be swapped with any other node. **E**. This multi-layer perceptron with uniform synaptic weights has 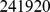 automorphisms(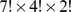) because each node of each layer can be swapped with any other node of the same layer.

Figure 8 shows the number of automorphisms for a few networks.

Each network has at least one automorphism, itself. The number of symmetry axes of a network therefore corresponds to the number of automorphisms minus one. Counting and enumerating automorphisms is a NP-complete problem, but there exist fast, exact algorithms that work for most graphs [43]–[45]. In the present work, we use the Bliss library [44].

We count the number of automorphisms of the developped neural network (and not those of the genotype). Modulatory neurons are labeled as “m” and other neurons as “n”. The Bliss library does not handle labeled edges, but edge labels can easily be transformed into node labels as follows: first, synaptic weights are binned into four categories (large negative, small negative, small positive, large positive); second, each of them is associated to a unique label; last, on each connection, a node is added and labeled by the category of the corresponding synaptic weight. Bias of neurons are ignored.

### Evolutionary algorithm

Networks are evolved using NSGA-II [47]. To mitigate premature convergence, we add to the fitness objective a *behavioral novelty objective* [4], [22], [23], [48], [49] that rewards individuals that do something that has not been done before. In effect, we transform the single-objective problem of maximimizing the fitness into a two-objective optimization problem:
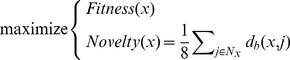
(5)where 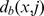 denotes the distance between the behaviors of individuals 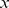 and 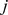, 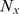 the set of the 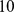 closest individuals to 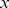 in the archive and the current population. At the end of each learning session (90 episodes), for each input pattern, the 4 outputs of the neural network are appended to a *behavior descriptor* (see the “Fitness function” section). The distance 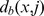 is the Euclidean distance between the behavior descriptors of 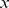 and 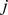.

### Parameters

#### Evolutionary algorithm (NSGA-II)

- population size: 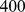

- number of generations: 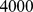

#### Plastic neuron model

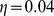

- maximum weight: 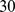

- minimum weight: 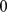 (no negative weight)
- maximum bias: 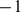

- minimum bias: 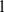

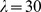

#### Softmax

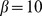

#### Direct encoding (control experiment, labeled graph, CPPN)

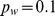
 (per connection)

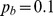
 (per neuron)

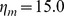
 (polynomial mutation)

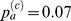

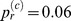

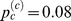

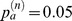

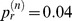

#### Map-based encoding

Each connection is labeled by a tuple 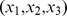:

- synaptic weight: 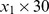

- inhibitory/excitatory: 
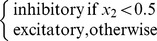

- connection type: 
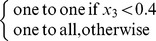

Each neuron is labeled by a tuple 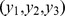:

- map/single neuron: 
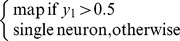

- modulatory/standard: 
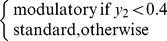

- bias: 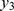

#### CPPN

- available functions: 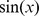, 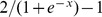 (sigmoid), 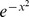 (Gaussian), 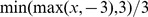 (linear)
- maximum connection weight: 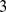

- minimum connection weight: 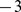

- other parameters: see direct encoding

#### HNN encoding

- threshold for the creation of a connection (LEO link): 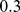

- synaptic weights are scaled to 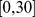

- modulatory vs standard: modularatory if output is below 0.4

### Source code

The source code for all the experiments is available at: http://pages.isir.upmc.fr/evorob_db

## Supporting Information

File S1 **List of the associations sets used in the experiments.**
(PDF)
